# Review of molecular biological research on the treatment of membranous nephropathy with Tripterygium glycosides based on TCM theory

**DOI:** 10.1097/MD.0000000000034686

**Published:** 2023-11-10

**Authors:** Pengyu Xu, Guangchu Fu, Haishen Zhao, Manya Wang, Hong Ye, Kejun Shi, Pin Zang, Xubo Su

**Affiliations:** a Shenzhen Pingle Orthopaedic Hospital/Shenzhen Pingshan District Hospital of Traditional Chinese Medicine/Shenzhen Orthopaedic Hospital, Shenzhen City, Guangdong Province, China; b The Fifth Affiliated Hospital of Southern Medical University, Guangzhou City, Guangdong Province, China; c Shanghai Pudong New Area Luchaogang Community Health Service Center, Shanghai, China.

**Keywords:** mechanism of action, membranous nephropathy, network pharmacology, TCM theory, Tripterygium wilfordii polyglycoside

## Abstract

To explore the mechanism of Tripterygium wilfordii polyglycoside (TWP) in the treatment of membranous nephropathy (MN) by network pharmacology. TCMSP and DrugBank databases were used to screen the main targets of the main active components of Tripterygium glycosides, and OMIM and Gene Cards databases were used to search the gene targets of MN. UniProt database was used to normalize all the targets to get the intersection targets of TGs and MNs. Synergistic genes were uploaded to the STRING platform to construct a protein-protein interaction network and screen related core targets. Gene Ontology and Kyoto Genome Encyclopedia analyses of core targets were performed using the DAVID database. AutoDockTools software was used to verify the molecular docking between the active components of TGs and the synergistic genes. We identified 126 potential targets for the active component of Tripterygium glycosides, 584 MN-associated disease targets, and 28 co-acting genes. It mainly involves AGE-RAGE signaling pathway, lipid and atherosclerosis, IL-17 signaling pathway, fluid shear stress and atherosclerosis, NF-kappa B signaling pathway and other pathways and biological pathways in diabetic complications. The active component of that Tripterygium glycosides and the active site of the synergistic core target can the bond energy is less than −5kJ/mol. Tripterygium glycosides can regulate the release of inflammatory factors to treat MN through multiple active components, multiple disease targets, multiple biological pathways and multiple pathways, which provides a basis for broadening the clinical use of traditional Chinese medicine in the treatment of MN.

## 1. Introduction

Membranous nephropathy (MN) is a kind of glomerular disease that can occur at any age with a variety of autoantigens to activate the immune response, damage the podocytes and induce proteinuria. Moreover, it is the most common cause of nephrotic syndrome in adults and one of the major risk factors for end-stage renal disease (ESRD).^[1]^ Most membranous nephropathy (MN) is an autoimmune disease, which is caused by the deposition of immune complexes in renal blood vessels leading to the thickening of glomerular capillary wall. According to the etiological classification, about 4 fifths of patients have no definite etiology and are called idiopathic membranous nephropathy. The other 20% of MN are often secondary to other diseases. Such as systemic lupus erythematosus, vasculitis, rheumatoid arthritis, viral hepatitis B and so on, called secondary membranous nephropathy. In addition, drugs, poisons, tumors or environmental factors can also cause secondary membranous nephropathy.^[2]^In the history of traditional Chinese medicine (TCM), there is no unified diagnosis of membranous nephropathy, but according to its clinical symptoms, it can be classified as “edema,” “turbid urine,” “consumptive disease” and other diseases. Modern Chinese medicine practitioners believe that the pathogenesis of MN is deficiency in origin and excess in superficiality, which is closely related to the 4 viscera of lung, spleen, liver and kidney. The pathogenesis of MN is based on the deficiency of spleen and kidney, and different pathological factors such as dampness, heat, phlegm and blood stasis may occur in different pathological stages. Therefore, attention should be paid to the spleen and kidney in the treatment, while taking into account the clearing of heat, eliminating dampness, promoting blood circulation and dredging collaterals.^[3]^

KDIGO IMN clinical practice guidelines recommend that the main treatment of MN is hormone combined with cyclophosphamide or calcineurin inhibitors, but this therapy has low effective rate, high recurrence rate, and is prone to complications such as infection and renal function damage.^[4,5]^Therefore, TCM has prominent advantages in the treatment of MN, such as improving serum albumin, reducing urinary protein, alleviating edema and fatigue, and has few adverse reactions, which has attracted more and more attention from physicians.^[6]^ Studies have found that Tripterygium glycosides have significant clinical efficacy and good safety in the treatment of MN, and significantly improve the quality of life of patients.^[7]^Tripterygium wilfordii is the root, leaf and flower of Celastraceae plant Tripterygium wilfordii. It was first recorded in Shen Nong Herbal Classic that it is pungent, warm in nature, bitter in taste, poisonous, and enters the liver and kidney meridians. It is recorded in Chinese Materia Medica that it has the effects of expelling wind and removing dampness, promoting blood circulation and dredging collaterals, detumescence and relieving pain, killing insects and detoxifying.Modern clinical studies have confirmed that Tripterygium wilfordii has anti-tumor, anti-inflammatory, immunosuppressive and other effects, mainly for the treatment of rheumatoid arthritis, systemic lupus erythematosus, nephrotic syndrome, allergic purpura, psoriasis and other refractory diseases, with significant clinical efficacy. Modern pharmacological studies have found that it has very obvious anti-inflammatory and immunosuppressive effects, and can be widely used in the treatment of inflammatory and immune diseases; a meta-analysis reported that Tripterygium wilfordii has a beneficial effect on the remission of idiopathic refractory nephrotic syndrome.^[8]^The Tripterygium glycosides (TGs) are extracted from the root of Tripterygium wilfordii, and their physiological activities are produced synergistically by a variety of components, including triptolide and celastrol.^[9]^ TGs not only retain the immunosuppressive effects of Tripterygium wilfordii crude drugs, but also remove many toxic components. Has immunosuppressive and anti-inflammatory effect, it has been proved that it can be used for the treatment of inflammatory and immune diseases.^[10]^ The MN rat model treated with TGs showed that it could significantly reduce the pathological renal injury of MN rat model, reduce the production of inflammatory cytokines, and improve oxidative stress, thereby significantly reducing the accumulation of less inflammatory injury and oxidative lesions.^[11]^The pathogenesis of MN is complex, which can be caused by a variety of endogenous and exogenous reasons, and there are many targets of TGs. Therefore, the specific mechanism and molecular biology of TGs in the treatment of MN are not clear.

TCM network pharmacology, as a new mode of modern TCM research and development, contains the holistic concept of TCM, including TCM unique diagnosis and treatment ideas and rich clinical experience, and constitutes a research mode characterized by “network and “system.”^[12]^Therefore, depending on the method of network pharmacology to mine the targets of TGs in the treatment of MN, and to verify the molecular docking, it will provide theoretical basis and experimental direction for the subsequent cellular and molecular experiments of TGs in the treatment of MN, and promote the clinical application of TGs for MN.

## 2. Materials and methods

### 2.1. Main databases and software

TCMSP, GeneCards, OMIM, Uniprot, Venny 2.1, Cytoscape v3.9.1, String Platform, DAVID database, RCSB PDB database, etc.

### 2.2. Discovery and screening of drug and disease targets

Targets of TGs were searched in TCMSP and DrugBank databases with the search terms “triptolide, tripterine,” and the species was limited to “Homo sapiens.” Through GeneCards, OMIM and other databases, the disease targets of MN were searched with the keyword “membranous nephropathy.”UniProt KB search function in UniProt database was selected, the database selection was limited to “Reviewed (Swiss-Prot)” and the species was “Human,” the targets were queried, and the above targets were normalized to obtain TGs and MN target sets.

### 2.3. Construction of protein-protein interaction network (PPI) between active components of TGs and potential disease targets of MN

The active components of TGs and potential disease targets of MN were introduced into the database of functional protein association networks (STRING), and the species was defined as Homo sapiens by Multiple proteins tool. Acquire a protein interaction, setting an interaction threshold to be a highest confidence “(>0.9), hide that free protein in the result, at the same time, save the PPI network as a TSV format file. The data were imported into Cytoscape _ v3.9.1 software for topological analysis of the network, and the protein-protein interaction network was drawn to screen the core proteins and obtain the core targets of the interaction.

### 2.4. Enrichment analysis of relevant action pathways

DAVID database was used for gene ontology analysis and kyoto encyclopedia of genes and genomes (KEGG) enrichment analysis of core targets. Biological Process, Cell Component and Molecular Function modules were selected for GO enrichment analysis. Pathway analysis was carried out through KEGG to obtain the pathways that were related to the pathogenic mechanism of MN and enriched with most of the key genes, and the data results were visualized.

### 2.5. Molecular docking to verify molecule-target relationship

TGs were used as ligands, and the potential core targets of TGs for MN were used as receptors for molecular docking. The 3-dimensional structure of TGs was downloaded from the Pubchem database, and the core target structure was downloaded from the RCSB PDB database. AutoDockTools 1. 5. 7 software was used to remove water molecules and ligands from the above files. At the same time, hydrogenation, electron addition and other operations were carried out, and the semi-flexible docking mode with default docking parameters was selected. The binding energy of the ligand molecule is <0 kJ/mol, indicating that the ligand molecule can spontaneously bind to the receptor protein. The binding energy is less than −5.0 kJ/mol, indicating that the binding energy is better, and the smaller the binding energy, the better the docking.

## 3. Results

### 3.1. Screening of active components of TGs and potential targets of MN

**Figure 1.**
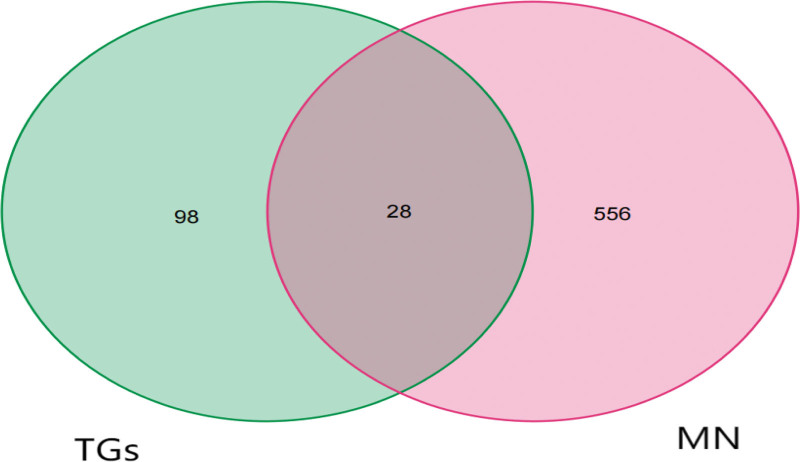
Cross-target of TGs and MN. Note: The red part is the TGs target gene, and the blue part is the MN target gene. MN = membranous nephropathy.

126 target genes of active components of TGs, 584 potential target genes of MN and 28 crossed target gene spots were screened through relevant databases (Fig. 1 and Table 1).

**Table 1:** TGs and MN cross targets.

Serial number	Uniprot ID	Protein name	Gene name	Degree
1	P10145	Interleukin-8	CXCL8	10.91
2	P14780	Matrix metalloproteinase-9	MMP9	10.883
3	P35354	Prostaglandin G/H synthase 2	PTGS2	10.882
4	P01375	Tumor necrosis factor	TNF	10.859
5	P15692	Vascular endothelial growth factor A	VEGFA	10.82
6	P40763	Signal transducer and activator of transcription 3	STAT3	10.637
7	P08253	72 kDa type IV collagenase	MMP2	10.595
8	P04637	Cellular tumor antigen p53	TP53	10.548
9	P01137	Transforming growth factor beta-1 proprotein	TGFB1	10.526
10	P42574	Caspase-3	CASP3	10.426
11	P05112	Interleukin-4	IL4	10.205
12	P03956	Interstitial collagenase	MMP1	10.183
13	P61073	C-X-C chemokine receptor type 4	CXCR4	10.149
14	P60568	Interleukin-2	IL2	10.05
15	P01579	Interferon gamma	IFNG	9.802
16	P42224	Signal transducer and activator of transcription 1-alpha/beta	STAT1	9.649
17	P01033	Metalloproteinase inhibitor 1	TIMP1	9.641
18	P25942	Tumor necrosis factor receptor superfamily member 5	CD40	9.562
19	P45983	Mitogen-activated protein kinase 8	MAPK8	9.167
20	P35968	Vascular endothelial growth factor receptor 2	KDR	9.117
21	P00749	Urokinase-type plasminogen activator	PLAU	8.408
22	P16035	Metalloproteinase inhibitor 2	TIMP2	7.75
23	P01024	Complement C3	C3	6.874
24	Q07812	Apoptosis regulator BAX	BAX	6.1
25	P08571	Monocyte differentiation antigen CD14	CD14	4.981
26	P10415	Apoptosis regulator Bcl-2	BCL2	4.228
27	Q02388	Collagen alpha-1 (VII) chain	COL7A1	2.675
28	P53420	Collagen alpha-4 (IV) chain	COL4A4	1.181

CXCL8 = interleukin-8, MMP2 = matrix metalloproteinase-2, MMP9 = matrix metalloproteinase-9, PTGS2 = prostaglandin G/H synthase 2, STAT3 = signal transducer and activator of transcription 3, TGFB1 = transforming growth factor beta-1 proprotein, TNF = tumor necrosis factor, TP53 = cellular tumor antigen p53, VEGFA = vascular endothelial growth factor A.

### 3.2. PPI network analysis and core target screening

**Figure 2.**
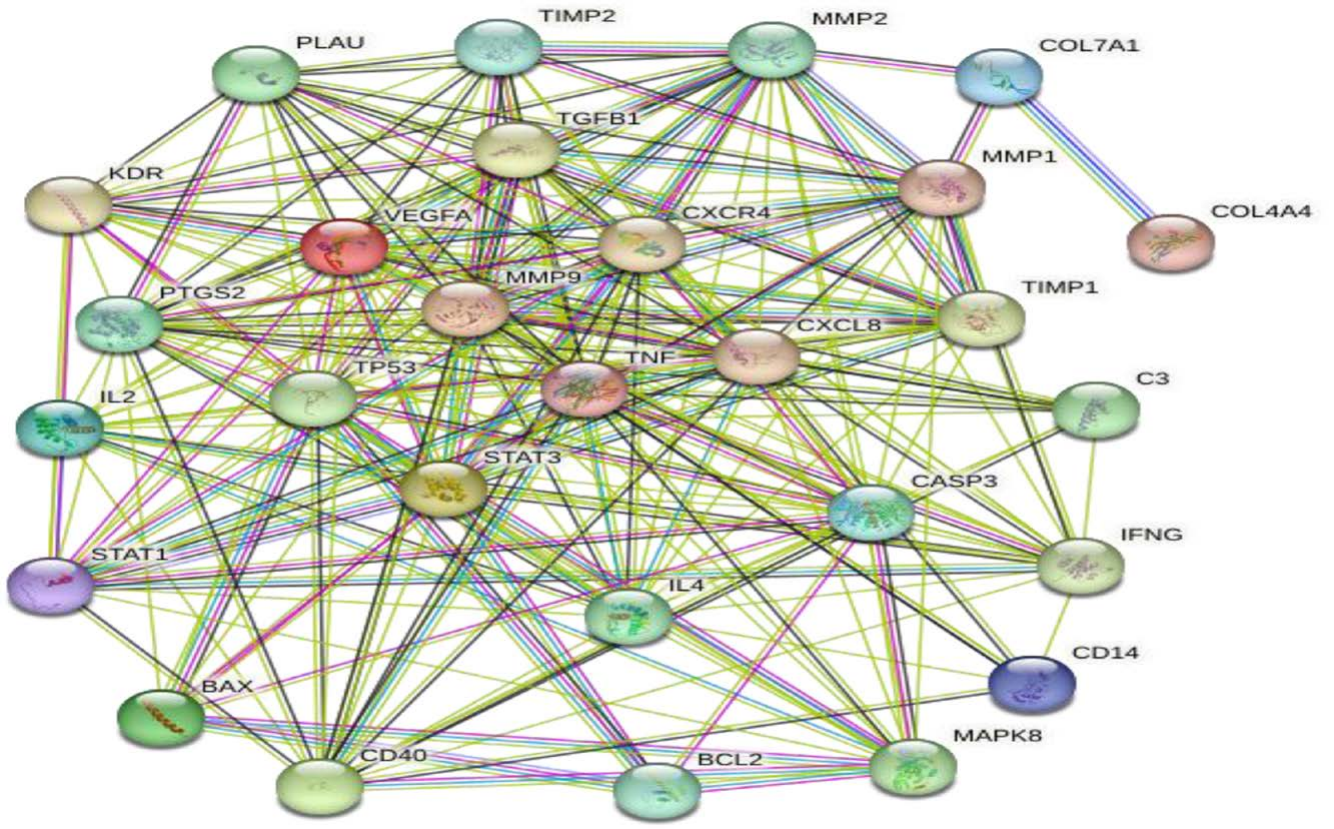
Intersected gene target PPI interaction network data. PPI = protein–protein interaction.

**Figure 3.**
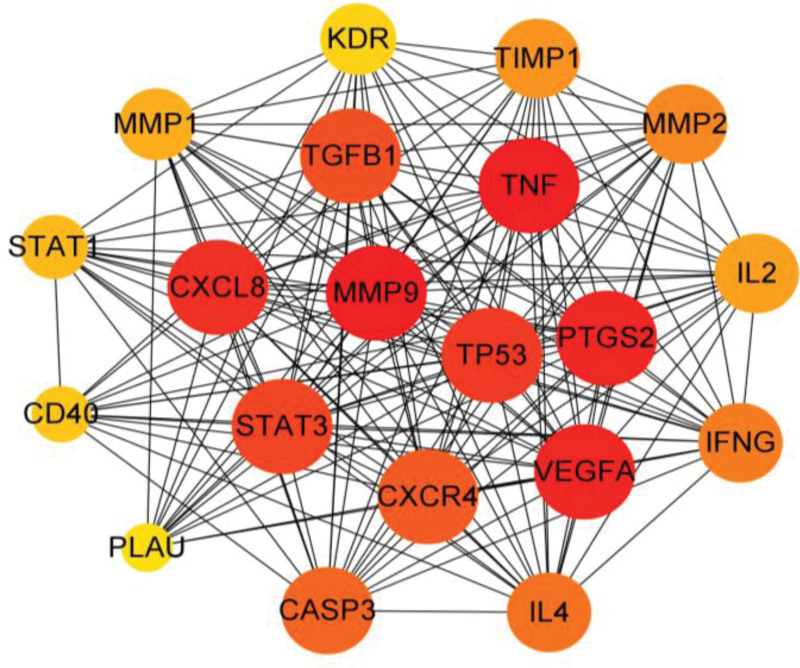
PPI network diagram of core gene target. PPI = protein–protein interaction.

The obtained 28 synergistic targets were imported into the String database to obtain PPI network data (Fig. 2). The PPI network data was imported into Cytoscape software for topological analysis, and 28 nodes and 462 edges were obtained. Meanwhile, the MCC algorithm of Cytohubba plug-in was used to process the data of synergistic targets, and the PPI network diagram was drawn for the top 20 synergistic targets with MCC values (Fig. 3).The larger the MCC value is, the closer the relationship between the drug and the target is. The color of the node represents the size of the MCC value, and the smaller the MCC value corresponding to the change of color from red to yellow. Therefore, the obtained 20 targets are used as the core targets for Jiaotai Pill to treat PI.

### 3.3. Visualization analysis of enrichment pathway of core gene target

**Figure 4.**
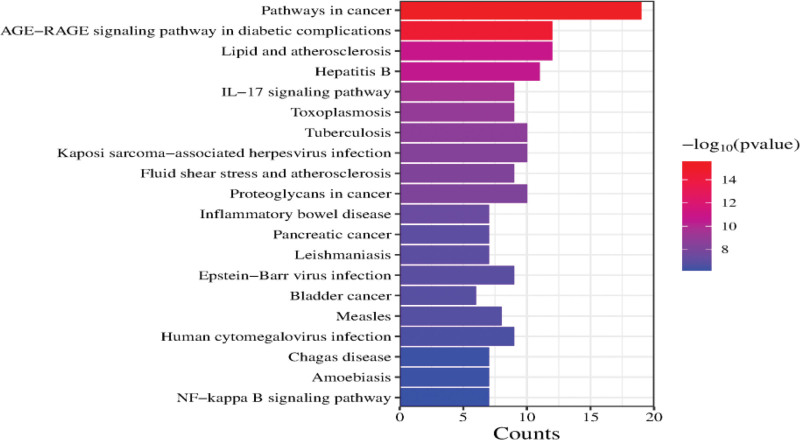
KEGG pathway analysis of core targets. KEGG = kyoto encyclopedia of genes and genomes.

**Figure 5.**
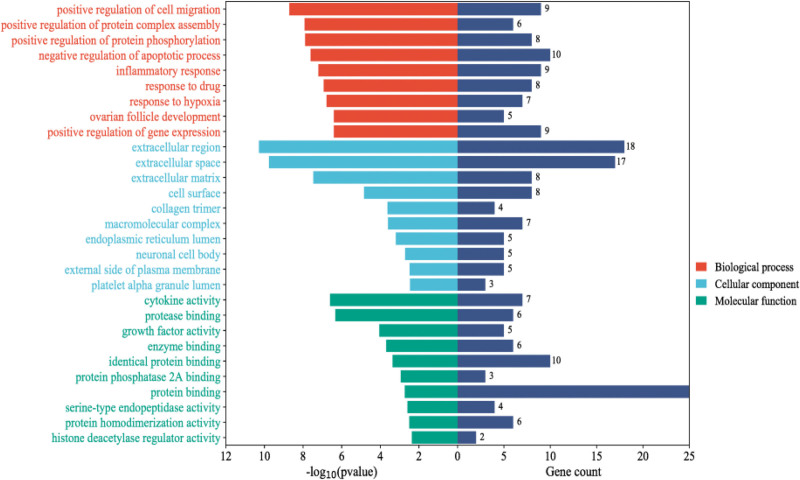
GO enrichment analysis of core action targets. GO = gene ontology.

The DAVID database was utilized for GO analysis and KEGG enrichment analysis of core targets, including biological processes, cellular components, and molecular functions as well as analysis via KEGG pathways. Through KEGG pathway analysis, the bar chart from red to blue represents the −Log10 (P) value from large to small; the length of the bar graph represents the number of genes enriched in the pathway, the horizontal axis represents the proportion of genes in the pathway, and the vertical axis represents the top 20 functional pathways enriched by genes. The results are shown in Figure 4. After GO analysis, the top 10 results with the highest −Log10 (P) values were selected for visual analysis, and the biological processes, cellular components, and molecular functions were plotted as bar graphs, with the length of the left bar graph representing the decreasing −Log10 (P) values and the length of the right bar graph representing the number of gene enrichments for the pathway. The results are shown in Figure 5.

### 3.4. Molecular docking of TGs with relevant core targets

The core target has a high degree of active sites and can form more than 2 hydrogen bonds, and the binding energy is less than −5 kJ/mol, so the core target has good binding activity. The binding energy of triptolide with PTGS2 was the lowest (−10.5 kJ/mol), and the binding activity was the strongest; while the binding energy of celastrol with MMP2 was the lowest (−9.9 kJ/mol), and the binding activity was better. TGs can form a good binding activity with the core target, which proves that TGs have outstanding performance in the treatment of MN. See Table 2 for details.

**Table 2:** Docking results of core target molecules.

PDB	Gene target	Binding energy of triptolide (kJ/mol)	Binding energy of celastrol (kJ/mol)
3IL8	CXCL8	−7.3	−6.3
1L6J	MMP9	−8.9	−8.5
5F19	PTGS2	−10.5	−9.5
1A8M	TNF	−8.4	−6.8
4KZN	VEGFA	−8.6	−6.6
6NJS	STAT3	−8.4	−7.8
1CK7	MMP2	−9.3	−9.9
1YCS	TP53	−6.4	−6.1
5VQP	TGFB1	−7.0	−7.5
1QX3	CASP3	−8.1	−7.7

CASP3 = Caspase-3, CXCL8 = interleukin-8, MMP2 = matrix metalloproteinase-2, MMP9 = matrix metalloproteinase-9, PTGS2 = prostaglandin G/H synthase 2, STAT3 = signal transducer and activator of transcription 3, TGFB1 = transforming growth factor beta-1 proprotein, TNF = tumor necrosis factor, TP53 = cellular tumor antigen p53, VEGFA = vascular endothelial growth factor A.

### 3.5. TCM treatment progress of MN based on syndrome differentiation

In recent years, great progress has been made in the study of TCM syndrome differentiation of large samples of MN. By analyzing the TCM prescription and clinical symptoms of adult MN, many medical scientists concluded that the TCM syndrome differentiation of adult MN was mainly based on “deficiency syndrome and excess syndrome.” Deficiency syndrome included deficiency of both qi and yin, deficiency of lung-spleen qi, deficiency of spleen-kidney Yang, and deficiency of liver-kidney yin; the syndromes of excess syndrome include blood stasis, rheumatism, retention of water and dampness, and retention of dampness and heat.^[13]^ In clinical practice, the most commonly used drugs for TCM treatment of MN include Tripterygium wilfordii Chinese patent medicines, Astragalus membranaceus Chinese patent medicines, Rehmannia glutinosa Chinese patent medicine, fermented Cordyceps sinensis powder Chinese patent medicine, and Salvia miltiorrhiza Chinese patent drugs, with significant efficacy and few adverse reactions.^[14]^A large number of clinical studies have shown that TCM is effective in the treatment of MN, with less side effects and high safety, and has achieved good clinical results. We summarized the randomized controlled trials (RCTs) of Tripterygium glycosides (LGT) in the treatment of MN, and found that LGT could improve the clinical efficacy of MN patients receiving basic treatment and significantly reduce the urinary protein content of patients. This study provides sufficient evidence-based medical evidence for TCM in the treatment of MN, and also lays the foundation for the promotion and application of TGs. See Table 3 for details.

**Table 3:** Randomized clinical trials of LGT in MN.

Study	Sample size (T/C)	Disease stage (T/C)	Intervention		Course of treatment	Outcomes (T/C)
			Treatment	Control		
Feng^[15]^	26/26	There were 10 cases in stage I, 16 cases in stage II, and 11 cases in stage I, and 15 in stage II.	LTG combined with Csa	Cyclosporine A	June	The total effective rate was 92.31%/69.23%.
Ling et al^[16]^	42/43	Phase I, Phase II, Phase III	LTG combined with valsartan	Valsartan	December	The total effective rate was 92.86%/74.42
Feng et al^[17]^	120/120	Phase I, Phase II, Phase III	LGT combined with low-dose prednisone acetate tablets	Low-dose prednisone acetate tablet	June	The total effective rate was 87.50%/54.17%.
Cui et al^[18]^	30/30	There were 8 cases in stage I, 12 in stage II, 10 in stage III/9 in stage I, 11 in stage II and 10 in stage III.	LGT in combination with prednisone and tacrolimus	Prednisone tablets combined with tacrolimus	June	The total effective rate was 93.3%/70.0%.
Mo et al^[19]^	32/33	1 case of stage I, 16 cases of stage II, 4 cases of stage III/2 cases of stage I, 16 cases of Stage II, 3 cases of Stage III	LGT combine with mycophenolate mofetil dispersible tablets and prednisone tablet	Mycophenolate mofetil dispersible tablets combined with prednisone tablet	June	The total effective rate was 90.48%/61.90%.
Qu et al^[20]^	28/28	19 in stage I, 9 in stage II/17 in stage I, 11 in stage II	LGT combined with losartan potassium tablets	Losartan potassium tablets	June	The total effective rate was 50.0%/17.9%.
Liu et al^[21]^	23/30	Phase I, Phase II, Phase III	LGT combined with prednisone	Tacrolimus combined with prednisone	September	The total effective rate was 90.0%/86.9%.

## 4. Discussion

Membranous nephropathy (MN) is one of the common causes of renal failure. Its clinical manifestation is nephrotic syndrome, with the highest incidence among people aged 30–50. Modern medical studies have confirmed that about 60–70% of cases of primary membranous nephropathy have autoantibodies against phospholipase A2 receptor (PLA2R), and MN is defined as an autoimmune disease.^[22]^Chinese scholars have found that the incidence of MN in different provinces in China is quite different. In Northeast China, MN has exceeded IgA, occupying the first place in primary glomerular diseases; the incidence in other areas of China is also increasing year by year.^[23]^

### 4.1. Analysis of core target action results

In this study, PPI network analysis screened out the core targets of TGs in the treatment of MN according to the value >10, and now the top 10 targets are listed, including CXCL8, MMP9, PTGS2, TNF, VEGFA, STAT3, MMP2, TP53, TGFB1, and CASP3. Jingyuan Xie et al Conducted a 9-cohort study involving 12820 individuals in East Asian and European populations. Genome-wide association studies of 4 East Asian cohorts with 4 841 individuals (1 632 primary MN cases and 3 209 controls) and 5 European cohorts with 7 979 individuals (2 150 primary MN cases and 5 829 controls) identified gene targets that were highly associated with MN. These include anti-phospholipase A2 receptor (PLA2R), nuclear factor NF-kappa-B p105 subunit (NFKB1), interferon regulatory factor 4 (IRF4), and human leukocyte antigen (HLA).^[24]^ At the same time, Jilin Chen et al^[25]^ conducted a retrospective cohort study and found that CXCL8 was highly correlated with the prognosis of MN;MMP9, MMP2 and TGFB1 are significantly expressed in patients with MN, which can be used as diagnostic markers of MN. MMP9 is significantly correlated with proteinuria, and MMP9 is significantly increased in patients with proteinuria in nephropathy^[26,27]^; Studies have found that glomerular inflammation in MN lesions has different signaling pathways, and PTGS2 and monocytes/macrophages are involved in the inflammatory process of nephritis^[28]^;Tumor necrosis factor (TNF), a pleiotropic cytokine with proinflammatory and immunomodulatory properties, has an important role in renal disease, which predicts renal progression in patients with MN^[29]^;Immunohistochemistry confirmed the high expression of VEGFA in MN renal biopsy specimens. VEGFA plays an important role in the pathogenesis of IMN through PI3K/AKT signaling pathway, providing a new target for the treatment of IMN^[30]^;Researchers found that CXCL12 in serum and urine of MN patients was significantly increased, and podocyte proliferation was inhibited in vitro induced podocyte injury model, CXCL12/CXCR4 and phosphorylated STAT3 (p-STAT3) were increased, and CXCL12/CXCR4 and p-STAT3 promoted cell proliferation. It can be used as a therapeutic target for MN patients^[31]^; upregulation of cell tumor antigen p53 (TP53) expression is closely related to renal pathological changes in MN^[32]^; CASP3 can reduce PLA2R activation and PI3K/AKT/mTOR pathway inhibition in PLA2R-activated podocytes, helping to protect podocytes from apoptosis.^[33]^

### 4.2. Analysis of KEGG pathway process

The core gene targets of this study were enriched in 102 KEGG pathways, and the results showed that the treatment of MN by TGs was highly related to the following pathways: diabetes-related, immune-related and cell survival-related pathways.

The diabetes-related pathway is the AGE-RAGE signaling pathway in diabetic complications and insulin resistance, which activates the NF-κB pathway and promotes the expression and release of IL6 and TNF, leading to cell activation and tissue damage.^[34]^In the podocytes of the kidney, the AGE-RAGE signaling pathway stimulates vascular endothelial growth factor, which causes proteinuria by increasing vascular permeability, and promotes the release of transforming growth factor-β1, leading to the production of glomerular extracellular matrix and tubular epithelial stroma^[35,36]^;The AGE-RAGE signaling pathway activates nicotinamide adenine dinucleotide phosphate oxidase, which in turn activates the signaling pathways mediated by mitogen-activated protein kinase, extracellular regulated protein kinase, extracellular signal-regulated kinase ERK1/2, and P38 through reactive oxygen species, and activates the NF-κB pathway through phosphorylation.^[37,38]^TGs can alleviate renal damage and reduce urinary protein levels in MN patients through AGE-RAGE signaling pathway. Immune-related pathways include Interleukin 17 (IL-17) signaling pathway, NF-κB signaling pathway, Th1, Th2 and Th17 cell differentiation, Toll-like receptor signaling pathway, T cell receptor signaling pathway and HIF-1 signaling pathway. IL-17 signaling pathway can activate NF-κB and induce NF-κB-dependent cytokines to up-regulate the expression of inflammatory genes;IL-17 has a pathogenic role in immune-mediated glomerular diseases.^[39]^ The activation of NF-κB signaling pathway can combine with other pathways, leading to renal inflammation and fibrosis.^[40]^ The HIF-1 signaling pathway is clearly upregulated when renal function is impaired by fibrosis.^[41]^TGs can treat MN by inhibiting renal local inflammation and hyperimmunity through IL-17 signaling pathway, Th1 and Th2 cell differentiation, toll-like receptor signaling pathway, NF-κB signaling pathway and HIF-1 signaling pathway. Cell survival related signaling pathways include TNF signaling pathway, apoptosis, NF-κB signaling pathway and IL-17 signaling pathway. The TNF signaling pathway induces podocyte apoptosis by inducing podocyte retinoic acid receptor responder 1 overexpression. While mediating renal inflammation and fibrosis, NF-κB signaling pathway also aggravates renal injury and proteinuria by inducing apoptosis through oxidative stress.^[42,43]^TGs can reduce renal cell apoptosis and autophagy, alleviate renal injury and reduce proteinuria by inhibiting the activation of signaling pathways such as TNF and NF-κB.

### 4.3. Summary and outlook

At present, there are few reports on the construction of drug-disease molecular networks in MN. Network pharmacology is helpful to further reveal the mechanism of TGs in the treatment of MN, to provide a direction for further study of the pharmacological and pharmacodynamic material basis of TGs in the treatment of MN, to provide a direction for the next target therapy, and to lay the foundation for the clinical promotion of traditional Chinese medicine. In this study, network pharmacology and molecular docking were used to predict the mechanism of action of TGs in the treatment of MN, and it was found that the mechanism was mainly related to the reduction of urinary protein and clinical symptoms by alleviating inflammatory response, immunosuppression and glomerulosclerosis.

However, in this study, the treatment of MN by TGs was theoretically verified, and a large number of clinical trials and related mechanism target studies were lacking. Therefore, further animal experiments are needed to verify its targets, which will lay a theoretical foundation for the next large sample, multi-center, randomized, double-blind and controlled clinical trials.

## Author contributions

**Conceptualization:** Pengyu Xu, Guangchu Fu.

**Data curation:** Haishen Zhao, Kejun Shi.

**Formal analysis:** Haishen Zhao, Kejun Shi.

**Funding acquisition:** Pengyu Xu.

**Investigation:** Pengyu Xu.

**Methodology:** Pengyu Xu, Manya Wang.

**Project administration:** Pengyu Xu, Manya Wang, Hong Ye, Xubo Su.

**Resources:** Manya Wang, Pin Zang.

**Software:** Pengyu Xu, Hong Ye, Pin Zang.

**Supervision:** Pengyu Xu, Hong Ye, Xubo Su.

**Validation:** Kejun Shi, Pin Zang.

**Visualization:** Xubo Su.

**Writing – original draft:** Pengyu Xu, Guangchu Fu.

**Writing – review & editing:** Pengyu Xu, Guangchu Fu.
